# Growth Inhibitory Activity of *Callicarpa americana* Leaf Extracts Against *Cutibacterium acnes*

**DOI:** 10.3389/fphar.2019.01206

**Published:** 2019-10-15

**Authors:** Rozenn M. Pineau, Sarah E. Hanson, James T. Lyles, Cassandra L. Quave

**Affiliations:** ^1^School of Biological Sciences, Georgia Institute of Technology, Atlanta, GA, United States; ^2^Center for the Study of Human Health, Emory College of Arts and Sciences, Atlanta, GA, United States; ^3^Department of Dermatology, Emory University School of Medicine, Atlanta, GA, United States; ^4^Emory University Herbarium, Atlanta, GA, United States

**Keywords:** medicinal plants, MIC, phytochemicals, acne, *Cutibacterium acnes*, biofilm, cosmeceutical

## Abstract

Acne vulgaris is a common skin disease affecting adolescents and young adults of all ethnic groups, negatively impacting self-esteem, self-confidence, and social life. The Gram-positive bacteria *Cutibacterium acnes* colonizes the sebum-rich follicle and contributes to inflammation of the pilosebaceous gland. Long-term antibiotic therapies targeting *C. acnes* lead to the development of antimicrobial resistance, and novel acne vulgaris therapies are needed. This study investigated the *C. acnes* inhibitory activity of *Callicarpa americana* leaves, a native Southeastern United States shrub historically used by Native Americans to treat fever, stomachache, and pruritis. Flash chromatography fractions of the ethyl acetate-soluble *C. americana* ethanol leaf extract (649C-F9 and 649C-F13) exhibited MICs ranging from 16 to 32 µg ml^−1^ and IC_50_ range of 4–32 μg ml^−1^ against a panel of 10 distinct *C. acnes* isolates. Cytotoxicity against an immortalized human keratinocyte cell line (HaCaTs) skin was detected at more than eight times the dose required for growth inhibitory activity (IC_50_ of 256 μg ml^−1^ for 649C-F9 and IC_50_ of >512 μg ml^−1^ for 649C-F13). This work highlights the potential of *C. americana* leaf extracts as a cosmeceutical ingredient for the management of acne vulgaris. Further research is necessary to assess its mechanism of action and *in vivo* efficacy.

## Introduction

### Pathogenesis of Acne Vulgaris

Acne vulgaris is a common skin disease affecting the vast majority of adolescents and a significant proportion of young adults. Approximately 85% of the population suffers from acne vulgaris at some point in their lives (Shah and Peethambaran, 2018). Acne negatively impacts self-esteem, self-confidence, and social life as it specifically affects the skin of the face, the chest, and the back—areas where the pilosebaceous gland concentration is the highest (Thomas, 2004; Titus and Hodge, 2012). Four processes characterize acne pathogenesis: 1) follicular hyperkeratinization, 2) excess of sebum, 3) colonization of *Cutibacterium acnes*, and 4) inflammation and immune response (Toyoda and Morohashi, 2001). *C. acnes* is a Gram-positive aero-tolerant anaerobic bacteria found in the sebaceous follicle. The oxygen-poor area formed by the lipid-rich obstructed follicle makes an ideal environment for the bacteria to proliferate. Other factors such as hormonal fluctuations or imbalance, stress, and pollution can also increase inflammation by providing a suitable environment for *C. acnes* growth (Bhambri et al., 2009).

### Current Treatment and Limitations

Current treatments for acne vulgaris include the use of topical antibiotics and/or chemical peeling agents. Treatments can also include daily oral antibiotics, retinoids, or hormones. In the United States, topical agents predominantly prescribed in the treatment of mild to moderate acne are composed of retinoids such as differin, tazorac, or retin-A, in combination with antibiotics such as clindamycin and erythromycin (Titus and Hodge, 2012). Benzoyl peroxide and salicylic acid are non-antibiotic therapies frequently added to creams to decrease the risk of developing antibiotic resistance and to reduce inflammation (Thiboutot et al., 2009). The most commonly used oral therapies include the tetracyclines (tetracycline, minocycline, and doxycycline), trimethoprim/sulfamethoxazole, and macrolides (erythromycin and azithromycin) (Leyden and Del Rosso, 2011).

A major disadvantage of current treatments is that daily intake of antibiotics places great selective pressure on bacteria to develop multidrug resistance (Van Den Bergh et al., 2016). Many countries have reported increasing resistance of *C. acnes* strains to topical macrolides (Walsh et al., 2016). Application of an active antibiotic gel increased resistance to erythromycin after only 12 weeks of treatment (Mills et al., 2002). The treatment of an individual’s acne can also have unintended effects on the microbiome, resulting in antibiotic resistance and proliferation of opportunistic pathogens elsewhere in the body. Margolis *et al.* reported that development of upper respiratory tract infections and pharyngitis was more likely in patients receiving oral antibiotics (Margolis et al., 2005; Margolis et al., 2012). In light of the growing concern for increasing antibiotic resistance of *C. acnes* and other bacteria due to antibiotic use in acne treatment, alternative therapies are urgently needed.

### Ethnopharmacological Relevance of *Callicarpa americana*

Ethnobotany is the study of plants used in different cultures, and it is a valuable framework from which to pursue drug discovery (Cox, 1994). Botanical therapies are a mixture of many active and non-active components, thus potentially having several modes of action, both direct and synergistically. The Quave Natural Product Library (QNPL) is a chemical library composed of over 1,900 plant extracts derived from 600 plant and fungal species, largely representing plants used in the traditional treatment of infectious and inflammatory skin disease. A screen of the QNPL against *C. acnes* revealed the growth inhibitory bioactivity of a crude leaf extract from *Callicarpa americana* L. (Lamiaceae), a native Southeastern American shrub. The *Callicarpa* genus has a rich history of use in traditional medicine and is characterized by the presence of biologically active terpenoids, such as monoterpenoids and diterpenoids (Kadereit, 2004). The fruits are the most striking elements of the plants in this genus—hence the genus name ‘*Callicarpa*,’ meaning “beautiful fruit,” and the common name, ‘Beauty Berry.’ The Alabama, Choctaw, Creek, Koasati, Seminole, and other Native American tribes used preparations of the roots, leaves, and branches for various medicinal purposes, including to treat fevers, stomachaches, and skin cancers (Taylor, 1940; Jones and Kinghorn, 2008).

Interestingly, *Callicarpa* species are also a key component of Asian traditional medicines (Tu et al., 2013). *C. arborea* Roxb. was traditionally used in India to heal cuts and wounds, and *C. tomentosa* Lam. was used to cure boils and eczema. In China, *C. macrophylla* Vahl, *C. pedunculata* R.Br., and *C. cathayana* C.H.Chang were applied to the skin to heal burns and bleeds (Jones and Kinghorn, 2008), highlighting the potential effectiveness of the *Callicarpa* genus to treat skin diseases (Gupta et al., 2010; Bhat et al., 2014).

### *Callicarpa americana* as a Source of Antimicrobial Therapies for Acne

Few studies have reported the bioactivity of the species *C. americana*. Three diterpenes (genkwanin, 16ξ-hydroxycleroda-3,13-dien-15,16-olide, and 2-formyl-16ξ-hydroxy-3-A-norcleroda-2,13-dien-15,16-olide) were isolated from the combined fruits, leaves, and twigs (fruiting branches) of *C. americana* and exhibit activity against human cancer cell lines (Jones et al., 2007). Intermedeol and callicarpenal (3,14,15,16-tetranorclerodane) compounds were found in essential oils from the leaves and acted as effective deterrents of *Aedes stephensi* and *Aedes aegypti* mosquitos (Cantrell et al., 2005). Research focused on other species within the same genus, and leaf extracts of *C. longifolia* Lam. in a gel preparation were recently found to exhibit antibacterial and wound healing effects on rabbit skin (Susilawati et al., 2018). A retinoid-containing gel in combination with a *C. nudiflora* Hook. & Arn. extract tablet was used in a clinical research study on acne vulgaris and was demonstrated to be more effective than the gel alone (Yang et al., 2010). These studies highlight the need for further research on *C. americana* extracts. In the present work, we demonstrate the promising growth inhibitory activity of *C. americana* on *C. acnes*.

## Materials and Methods

### Plant Collection and Identification

Fresh leaves of *C. americana* were harvested from wild populations in Atlanta, GA, in June and August 2017. Voucher specimens (**Table 1**) were deposited at the Emory University Herbarium (GEO), and species identity was confirmed by botanist Tharanga Samarakoon, Ph.D. Specimens were digitized and are available for viewing on the SERNEC portal (Sernec, 2019). Bulk plant material was dried in a dehumidification chamber and then ground into a fine powder through a 2-mm mesh with a Thomas Scientific Wiley Mill (Swedesboro, NJ). Retention vouchers of dried and ground material were prepared for future reference and stored in The Quave Research Laboratories at Emory University.

**Table 1:** List of *Callicarpa americana* specimens harvested from wild populations in Atlanta, GA.

GEO accession number	Collection date	Collection location	Location coordinates
22044	June 26, 2017	Hahn Wood, Emory University; USA, Georgia, DeKalb County	33.8036944440 N, −84.3230833330 W
22204	Aug. 11, 2017	Coralwood Centre School; USA, Georgia, DeKalb County	33.8281800000 N, −84.2872430000 W
22205	Aug. 11, 2017	Coralwood Centre School; USA, Georgia, DeKalb County	33.8281800000 N, −84.2872430000 W

Voucher specimens of all populations harvested were collected and deposited at the Emory University Herbarium (Index Herbariorum Code: GEO). Digitized specimens are available for viewing online through the SERNEC portal (Sernec, 2019).

### Preparation of Extracts

Dry, ground plant biomass was double macerated in 95% ethanol at a 1:10 ratio (*w*/*v*) for 72 h of maceration with daily agitation. The liquid was then decanted and vacuum filtered, then concentrated by rotary evaporation at ≤40°C. Extracts were redissolved in dH_2_O, shell frozen in a dry ice–acetone bath, and lyophilized overnight on a Labconco FreeZone 2.5 Lyophilizer (Kansas City, MO). Dry crude extract (Extract ID: 649) was scraped into scintillation vials and stored at −20°C until microbiological testing, at which time they were dissolved in DMSO to a stock concentration of 10 mg ml^−1^.

### Fractionation Strategy

**Figure 1:**
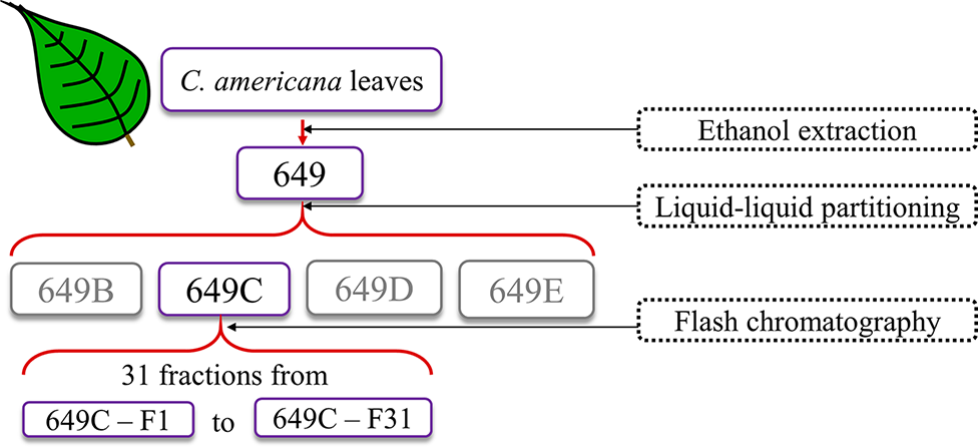
Extraction and fractionation strategy for *C. americana* dried leaves.

The crude extract (649) was partitioned against hexanes, ethyl acetate, *n*-butanol, and water following a modified Kupchan scheme. Subsequently, the ethyl acetate partition (649C) was determined to be the most active partition by antimicrobial bioassays and was further fractionated *via* flash chromatography. The full fractionation strategy is detailed in **Figure 1**.

**Figure 2:**
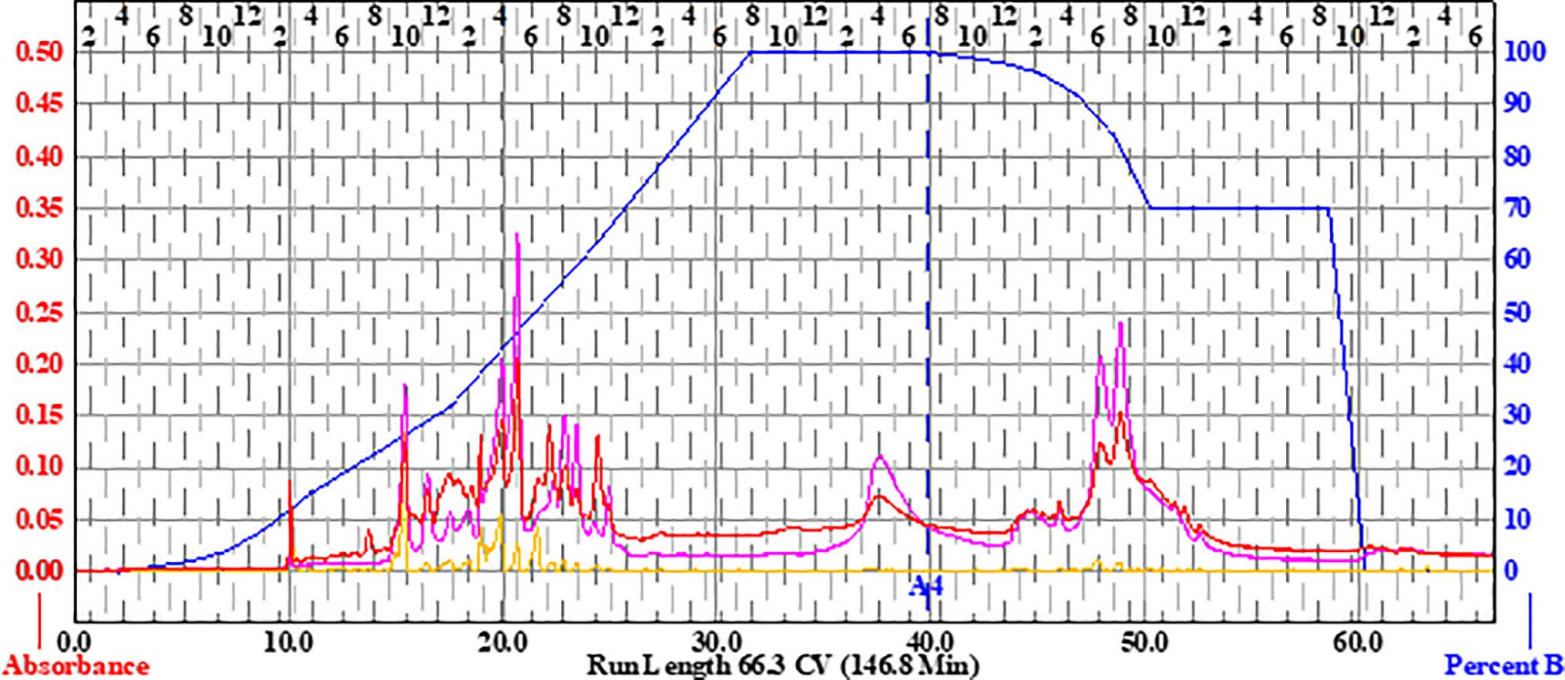
Flash chromatogram of 649C fraction. A 330-g Gold Silica column was loaded with 15.36 g of the 649C fraction. Three mobile phases— hexanes, ethyl acetate, and methanol—were used to perform the separation. The blue line details the percent of ethyl acetate mobile phase. In total, 31 fractions were decided based on the chromatogram, with each peak being assigned to a unique fraction if possible. Absorbance of the extract is read on the *left axis* (*red*, *orange*, and *pink lines* for three wavelengths: 254 nm, 320 nm, and total absorbance of 200–780 nm, respectively). The *x-axis* corresponds to column volumes (CV) with a total run length of 66.3 CV.

Briefly, to prepare the sample, 15.36 g of the extract was dissolved in methanol, Celite was added at a 1:4 ratio (*w*/*w*), the mixture was concentrated *in vacuo*, and the dry preparation was loaded into a Teledyne solid-phase load cartridge. A 330-g RediSep Rf Gold Silica column was used with the CombiFlash Rf+ (Teledyne ISCO) flash chromatography system. Three mobile phases—hexanes, ethyl acetate, and methanol—were used to perform the separation (**Table 2** and **Figure 2**). The resulting tubes were combined into 31 fractions, dried *in vacuo*, lyophilized, scraped into scintillation vials, and then stored at −20°C until testing.

**Table 2:** Gradient table for the flash chromatography separation of extract 649C.

Minutes	% Hexane	% Ethyl Acetate	% Methanol
0	100	0	0
4.4	100	0	0
8.2	99	1	0
12	98	2	0
15.8	96	4	0
19.6	91.9	8.1	0
24.3	85.4	14.9	0
38.9	68.1	31.9	0
54.3	35.9	641	0
69.8	0	100	0
88.2	0	100	0
88.4	0	100	0
92.2	0	99	1
96	0	98	2
99.8	0	96	4
103.6	0	92	8
107.4	0	84	16
111.2	0	70	30
129.7	0	70	30
133.3	0	0	100
146.6	0	0	100

The flow rate for the 330-g RediSep Rf Gold Silica column was 200 ml min^−1^.

### Antibacterial Testing

#### Bacterial Strains and Cultures

Extracts were first tested against a *C. acnes* isolate obtained from the American Type Culture Collection (strain ATCC6919). Based on these results, the most active fractions and their parent extracts were tested for growth inhibitory activity in nine additional clinical isolates of *C. acnes*; strain and source details are provided in **Table 3**. All bacteria were streaked onto tryptic soy agar (TSA) plates supplemented with 5% sheep blood (Hardy). Plates were incubated at 37°C until individual colonies were visible (72 h for most isolates and 120 h for the slowest growers) before individual colonies were transferred into Brain Heart Infusion broth supplemented with 1% dextrose (BHId). Liquid cultures were incubated for an additional 72 h before preparation of a working culture for biological assays. An EZ Anaerobe Chamber and GasPak EZ Anaerobe (BD) container sachets were used to create the anaerobic conditions required for *C. acnes* growth.

**Table 3:** *C. acnes* strain details.

Strain ID	Strain genotype	Type of isolate	Body site
ATCC6919	–	Laboratory strain	Facial acne
B104.1	Ribotype 3	Clinical isolate	Nasal microcomedone
B104.2	Ribotype 3	Clinical isolate	Nasal microcomedone
B104.4	Ribotype 3	Clinical isolate	Nasal microcomedone
B104.5	Ribotype 3	Clinical isolate	Nasal microcomedone
B104.6	Ribotype 1	Clinical isolate	Nasal microcomedone
B104.7	Ribotype 1	Clinical isolate	Nasal microcomedone
B104.8	Ribotype 3	Clinical isolate	Nasal microcomedone
HL013PA1; HM-497; EU-09	–	Clinical isolate	–
HL030PA1; HM-504; EU-16	–	Clinical isolate	–

Clinical isolates B104.1 to B104.8 were obtained from Dr. Laura Marinelli (Marinelli et al., 2012). Clinical isolates EU-09 and EU-16 were obtained through BEI Resources, NIAID, NIH as part of the Human Microbiome Project.

– indicates unknown information from the strain providers.

#### Growth Inhibition Assays

As no Clinical and Laboratory Standards Institute (CLSI) guidelines for MIC testing in *C. acnes* have been published, we followed a previously described microtiter broth method (Nelson et al., 2016). Liquid cultures were standardized to an optical density of 0.05 at 590 nm (OD_590nm_), which corresponds to 5 × 10^7^ CFU ml^−1^, using a Cytation-3 multimode plate reader (BioTek). This was further confirmed by plate counts. BHId was used for adjusting the final inoculum density.

The growth inhibition assay was performed in a sterile 96-well plate (Falcon 351172) in a total well volume of 200 µL. The dose tested ranged from 0.125 to 64 μg ml^−1^ for ATCC6919 and from 2 to 64 μg ml^−1^ for all other clinical isolates. Negative controls (no treatment and DMSO vehicle treatment), positive control (clindamycin treatment), and a media control (BHId alone) were included, and all treatments were conducted in triplicate and repeated on two separate days. A first OD_600nm_ determination was conducted immediately following plate setup, prior to a 72-h incubation period at 37°C in an anaerobe chamber. A second OD_600nm_ determination was taken 72 h post-inoculation, and the final percent inhibition of growth was determined following a previously reported formula, which takes into account the effect of the extract color (Quave et al., 2008). The IC_50_ and MIC are the minimal concentrations to inhibit at least 50% and 90% of the growth, respectively.

#### Biofilm Eradication

Liquid cultures were standardized to an optical density at 590 nm (OD_590nm_) of 0.05, which corresponds to 5 × 10^7^ CFU ml^−1^, and pipetted into a 96-well tissue culture plate (TPP92097). After a 24-h incubation period at 37°C in an anaerobic chamber, the media was gently removed using a single-channel micropipette without disturbing the biofilm. Fresh BHId and treatment was added to each well. The plate was incubated for an additional 24 h at 37°C in an anaerobe chamber. The media was gently removed before fixing the biofilms in two steps: adding 100% ethanol to each well, removing it, and then heat fixing at 60°C for 60 min. Crystal violet was then added to each well for 5 min to stain the biofilms and was subsequently gently rinsed out under tap water. Plates were dried overnight, then a solution of 2.5% Tween 80 in ethanol was added to elute the stain for 15 min, the eluent transferred to a new 96-well plate, and an OD_595nm_ obtained to determine total biofilm mass in each well. The minimal biofilm eradication concentration (MBEC_50_) was defined as the minimal concentration of extract required to eradicate at least 50% of the preformed biofilm.

### Mammalian Cytotoxicity Assay

Mammalian cytotoxicity of extracts was assessed using human keratinocytes (HaCaTs) and a lactate dehydrogenase (LDH) test kit (G-Biosciences, St. Louis, MO) as previously described (Quave et al., 2015). Extracts were sterile-filtered with 0.2-µm syringe filters and tested at a concentration range of 2–1024 μg ml^−1^. Percent DMSO (*v*/*v*) in the wells was <2% for all tests.

### Chemical Characterization

#### LC-FTMS Analysis

Liquid chromatography–Fourier transform mass spectrometry (LC-FTMS) was performed on 649C fractions using a Shimadzu SIL-ACHT (Columbia, MD) and Dionex (San Jose, CA) 3600SD HPLC pump. The stationary phase was a Phenomenex Kinetex C18 150 . 2.1 mm, 2.5 μm with guard column at room temperature. Mobile phases were Optima LC/MS (Fisher Scientific, Waltham, MA) consisting of (A) 0.1% formic acid in water (B) and 0.1% formic acid in methanol at a flow rate of 0.15 ml/min. The gradient profile consisted of initial conditions of 70:30 A/B which was held until 2.6 min., then a linear gradient applied until 5:95 A/B was reached at 52.7 min—this was held until 61.6 min—then a step gradient applied to 0:100 A/B, which was held until 70.4 min, and then the column returned to initial conditions prior to the next injection. A 20-μl injection of each fraction was applied onto the column. The chromatography was monitored at 190–600 nm by a Dionex DAD detector. The MS data was acquired in MS^1^ mode scanning a *m*/*z* of 150–1,500 on a Thermo Scientific LTQ-FT Ultra MS in positive ESI mode and processed with Thermo Scientific Xcalibur 2.2 SP1.48 software (San Jose, CA). The capillary temperature was 275.0°C, sheath gas of 40, source voltage and current of 5.0 kV and 100.0 μA, and capillary voltage of 29.0 V.

Putative formulas and compounds were determined for peaks of the bioactive fractions of 649C with greater than 1% relative abundance by area in the MS total ion chromatograph (TIC). Scifinder (Chemical Abstracts Service) was searched in July 2019 to identify putative matches. The (M+1)^+^ from the MS data was used to calculate the accurate mass of the parent ion and the databases searched for small molecules from the genus *Callicarpa* within ±1.0 Da. The molecular formulas of the hits were compared to empirical formulas derived from the experimental MS data. Database hits that matched the experimentally calculated empirical formula, ± 10 ppm, were evaluated further. Publications on the remaining small molecules were reviewed and the presence of the compound in the genus was verified. The resulting putative compound matches are in **Table 5**. Only an empirical formula is reported for ions that do not have a match in the database.

## Results

### Growth Inhibitory Activity Against *P. acnes*

**Figure 3:**
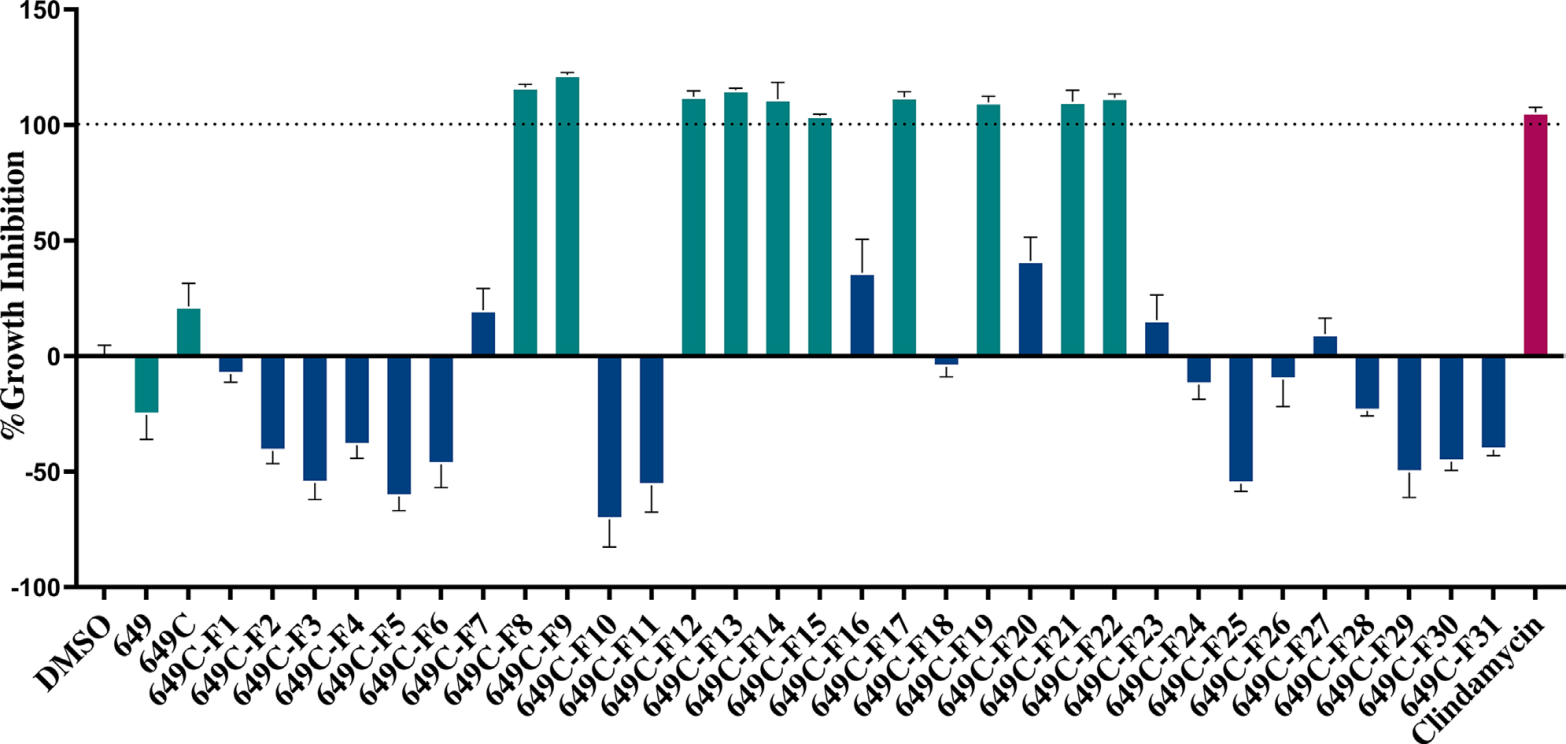
Fractions of 649C were screened at 64 µg ml^−1^ to determine which ones exhibited the most growth inhibitory activity against *C. acnes* strain ATCC6919. Fractions exhibiting >90% growth inhibition and the parent extracts 649 and 649C (*green bars*) were further tested for growth inhibition dose–response assay, biofilm eradication, and cytotoxicity against human skin cells. The *blue bars* represent the least active fractions. Clindamycin was used as a positive antibiotic control (*pink bar*).

**Figure 4:**
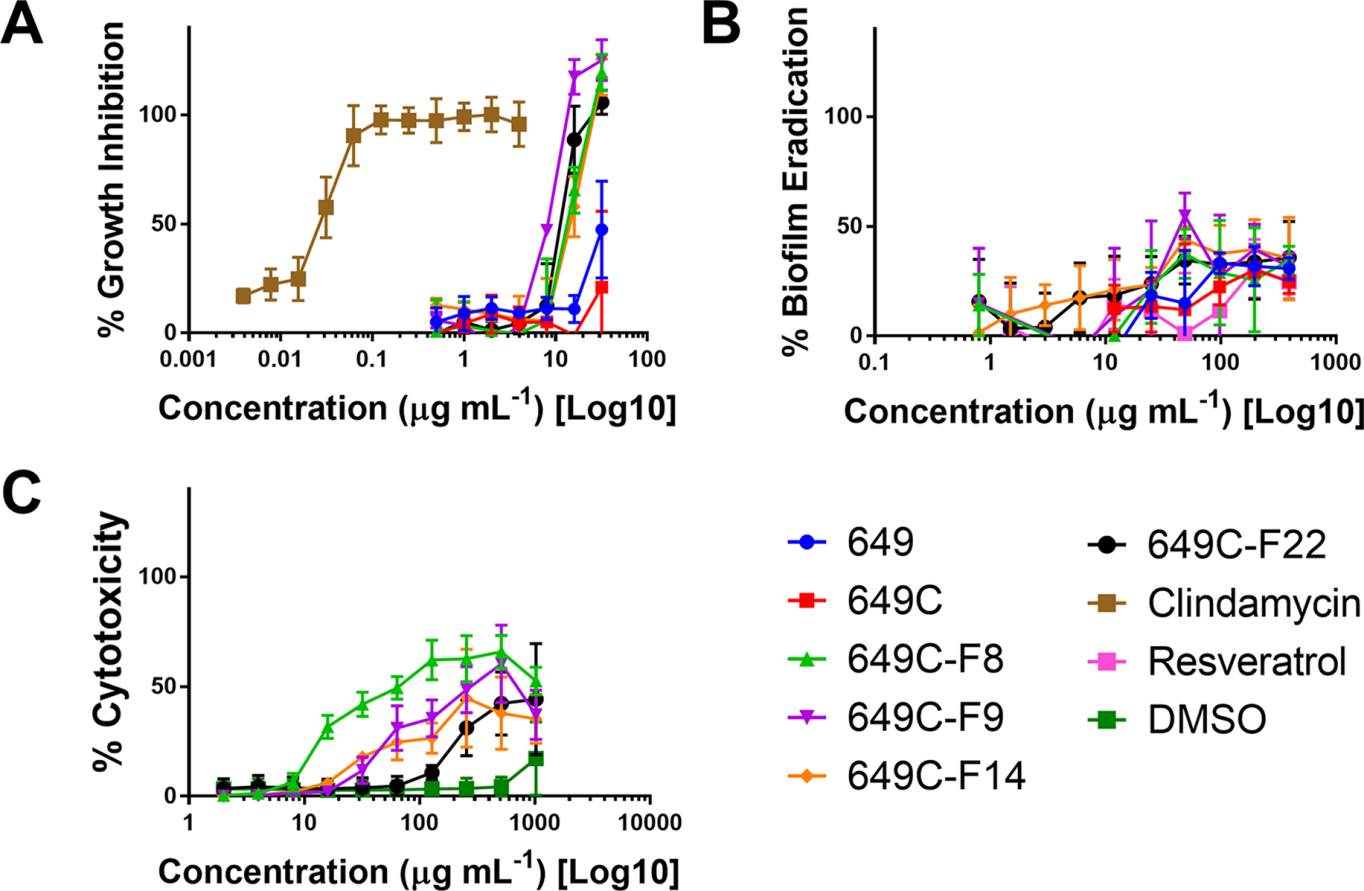
**(A)** Growth inhibitory activity, **(B)** biofilm eradication, and **(C)** cytotoxicity on human keratinocytes (HaCaTs) of *C. americana* crude extract and the most active fractions. Tested fraction concentrations ranged from 0.25 to 64 µg ml^−1^, from 0.5 to 394 µg ml^−1^, and from 2 to 1,024 µg ml^−1^ for growth inhibition, biofilm eradication, and cytotoxicity assays, respectively. *Points* represent means and *bars* are standard error of the mean. *Dotted lines* represent 50% and 90% thresholds.

**Figure 5:**
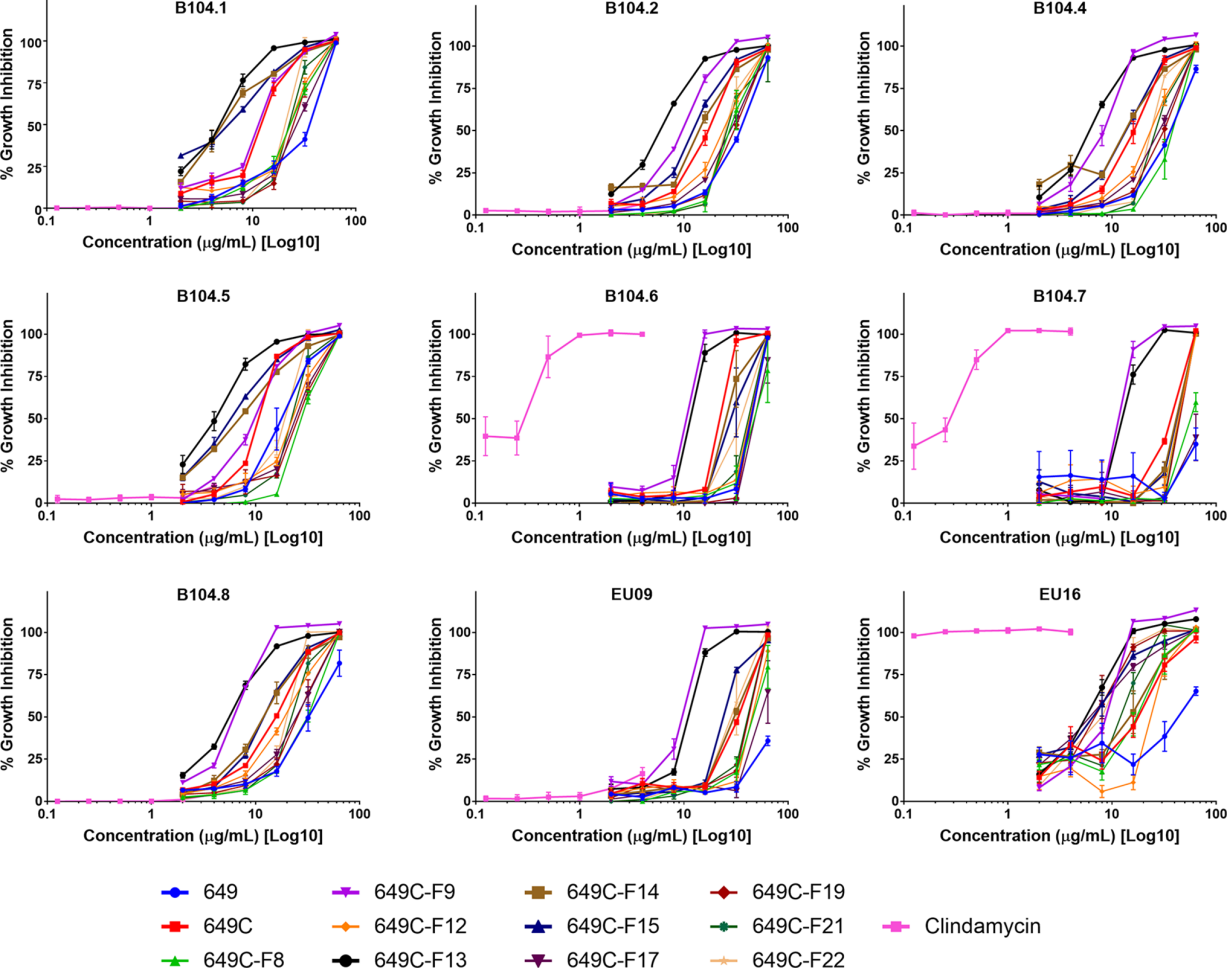
Growth inhibitory activity of extracts and fractions against a panel of nine clinical isolates of *C. acnes*.

**Figure 6:**
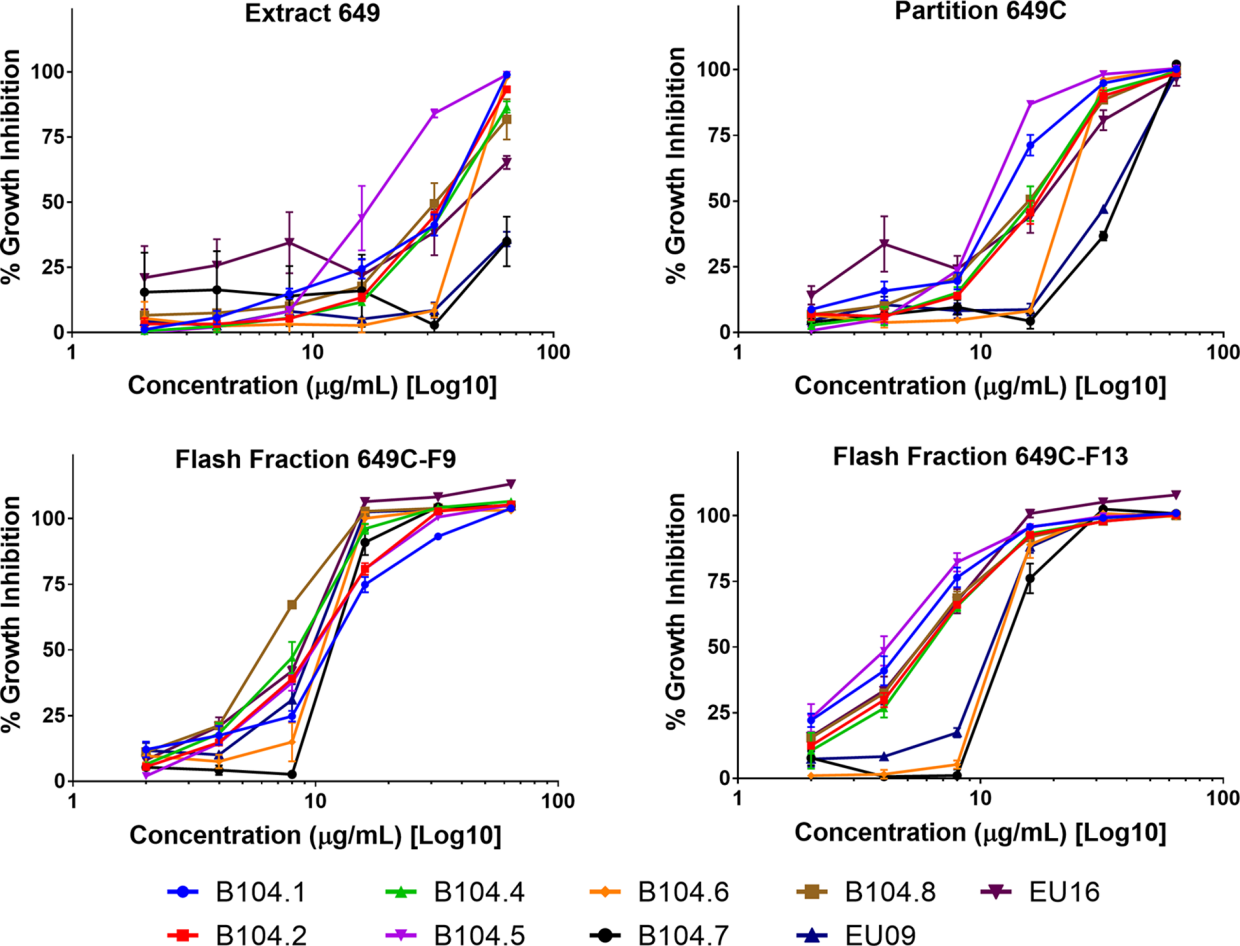
Growth inhibitory activity of the most active fractions and their parent extracts against a panel of nine clinical isolates of *C. acnes*.

Growth inhibitory effects of the crude extract and partitions of *C. americana* leaves were determined using a static MIC assay. The initial screen was performed on ATCC6919 and demonstrated that the ethyl acetate partition (649C) was the most effective inhibitor of *C. acnes* growth with an IC_50_ value of 32 μg ml^−1^. 649C was subjected to flash chromatography to yield 31 fractions. Eight out of the 31 fractions exhibited 100% growth inhibition at 64 μg ml^−1^ against ATCC6919 (**Figure 3**). Dose-dependent growth inhibition of the most active fractions was then conducted on ATCC6919 as well as nine other clinical isolates of *C. acnes* (**Figures 4** and **5**). Growth inhibitory activity was observed for each of the 10 isolates of *C. acnes* tested. The most active fractions were 649C-F9 and 649C-F13, with IC_50_ values ranging from 4 to 32 μg ml^−1^ and MIC values ranging from 16 to 32 μg ml_-_^1^ across all isolates (**Table 4** and **Figure 6**). Although no interpretive standards from the CLSI for antibiotic resistance exist for *C. acnes*, it is noteworthy that seven of the nine clinical isolates tested exhibited MICs >4 μg ml^−1^ for the antibiotic control (clindamycin), which is commonly used in both oral and topical therapies for the management of acne vulgaris. Furthermore, six of the nine clinical isolates tested did not achieve even 50% growth inhibition at the maximum concentration of clindamycin tested (4 μg ml^−1^).

**Table 4:** *C. americana* extract, partition, and fraction growth inhibition (IC_50_ and MIC) and HaCat cytotoxicity IC_50_ values (in µg ml^−^^1^).

		649	649C	649C-F8	649C-F9	649C-F12	649C-F13	649C-F14	649C-F15	649C-F17	649C-F19	649C-F21	649C-F22	Cli-
**IC_50_ for *C. acnes*** **growth**	**ATCC6919**	32	32	16	8	16	16	16	16	16	16	16	16	0.0625
	**B104.1**	64	16	32	16	32	8	8	8	32	32	32	32	>4
	**B104.2**	64	32	32	32	32	8	16	16	32	32	32	32	>4
	**B104.4**	64	16	64	8	32	8	16	16	32	32	32	32	>4
	**B104.5**	32	16	32	16	32	4	8	8	32	32	32	32	>4
	**B104.6**	64	32	64	16	64	16	32	32	64	64	64	64	0.5
	**B104.7**	>64	64	64	16	64	16	64	64	>64	64	64	64	0.5
	**B104.8**	64	16	32	8	32	8	16	16	32	32	32	32	>4
	**EU-09**	>64	32	64	16	64	16	32	32	64	64	64	32	>4
	**EU-16**	64	32	32	16	32	8	16	8	8	8	16	16	< 0.125
**MIC for *C. acnes*** **growth**	**ATCC6919**	>32	>32	32	16	16	16	32	32	16	16	16	16	>8
	**B104.1**	64	32	64	32	64	16	32	32	64	64	64	64	>4
	**B104.2**	64	32	64	32	64	16	64	32	64	64	64	64	>4
	**B104.4**	64	32	64	16	64	16	64	32	64	64	64	64	>4
	**B104.5**	64	32	64	32	64	16	32	32	64	64	64	32	>4
	**B104.6**	64	32	>64	16	64	16	64	64	>64	64	64	64	0.5
	**B104.7**	>64	64	>64	16	64	32	64	64	>64	64	64	64	1
	**B104.8**	>64	32	64	16	64	16	32	32	64	64	64	32	>4
	**EU-09**	>64	64	>64	16	64	16	64	64	>64	64	64	64	>4
	**EU-16**	>64	64	32	16	64	16	64	32	32	16	32	16	0.125
**HaCat**	**IC** **_50_**	> 1024	> 1024	128	256	>512	>512	> 1024	> 512	512	>512	>512	1024	–
**TI**	**Lower range**	16	32	2	8	8	32	16	8	8	8	8	16	–
	**Upper range**	>32	>64	8	32	32	128	128	64	64	64	32	64	–

All values were determined as compared to the vehicle control for at least three replicates. The therapeutic index (TI) is reported as a range of values for the 10 C. acnes isolates (cytotoxicity IC_50_ divided by growth inhibition IC_50_).

Cli clindamycin, antibiotic control.

### Biofilm Eradication Activity in *C. acnes*

Biofilm eradication activity was assessed, and none of the fractions were found to significantly eradicate the biofilm. A decrease in 30–40% of biofilm was observed for the highest concentration tested (394 μg ml^−1^). Resveratrol, a previously reported candidate for *C. acnes* biofilm eradication, reached 30% eradication at a concentration of 394 μg ml^−1^.

### *C. americana* Extracts Exhibit Low Toxicity to Human Keratinocytes

To examine possible cytotoxic effects of the extracts in mammalian cells, HaCaT cells were treated at concentrations ranging from 2 to 1,024 μg ml^−1^. For all tested fractions, cytotoxicity (IC_50_) was only observed at more than eight times the dose required for inhibitory activity when compared to the IC_50_ values for *C. acnes* strain ATCC6919 (**Table 4**). Fractions 649C-F8 and 649C-F9 were the most cytotoxic to the HaCaTs, with IC_50_ values at 28 and 256 μg ml^−1^, respectively. The therapeutic index (TI) was calculated by dividing the IC_50_ for mammalian cytotoxicity by the IC_50_ for *C. acnes* growth inhibition, and the TI range for each fraction is reported in **Table 4**.

### Chemical Characterization of Extracts

**Figure 7:**
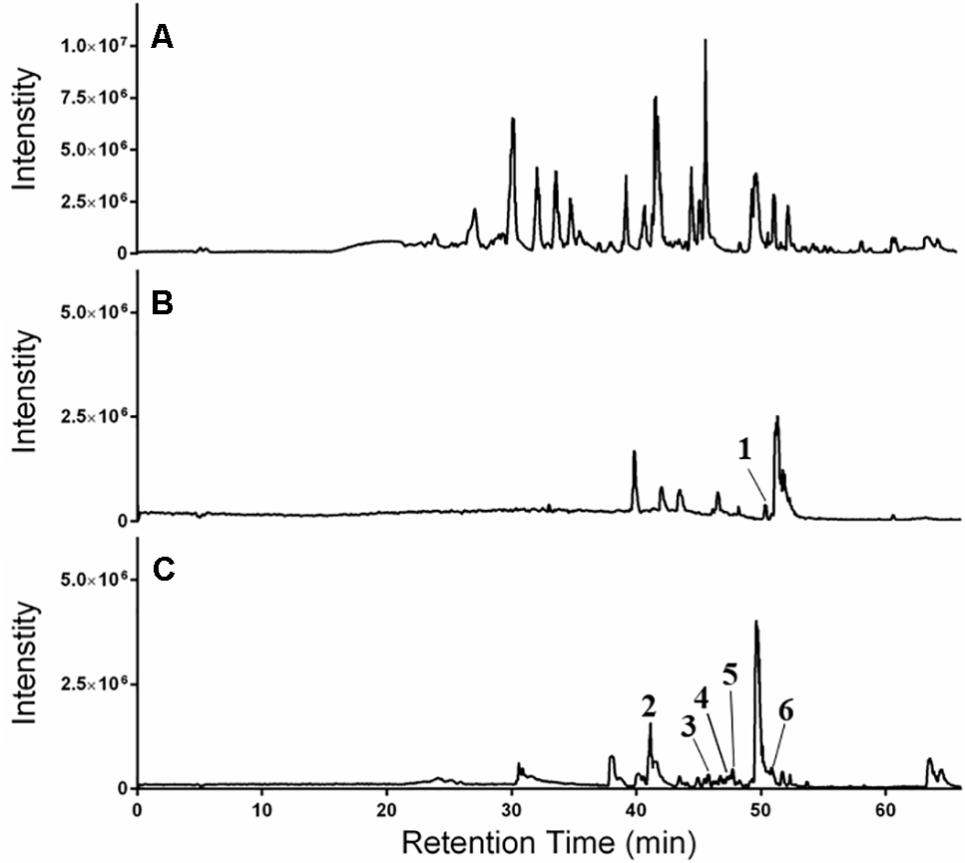
HPLC-FTMS ESI-positive chromatograms for **(A)** 649C, **(B)** 649C-F9, and **(C)** 649C-F13. Peaks **1**–**6** are indicated.

The LC-FTMS ESI-positive mass spectral chromatograms of the ethyl acetate partition (649C) and the bioactive fractions (649C-F9 and 649C-F13) are shown in **Figure 7**. The ions with greater than a 1% abundance in the LC-FTMS ESI-positive mode analysis are tabulated in **Table 5**. Fraction 649C-F9 is the least complex fraction, with over 56% consisting of a compound with *m*/*z* 637.4457 eluting at 51.3 min. A total of nine ions were identified as having greater than 1% relative abundance. Putative matches were only obtained for peak number **1** with an empirical formula of C_20_H_29_O_3_, which corresponded to 14 compounds in Scifinder: 16ξ-hydroxycleroda-3,11(E),13-trien-15,16-olide (CAS #**935527-99-4**), seco-hinokiol (**834870-61-0**), angustanoic acid F (**104998-60-9**), 7-hydroxydehydroabietic acid (**95416-24-3**), 11-hydroxylsugiol (**88664-08-8**), 3,4-dihydroxyphenethyl glucoside (**76873-99-9**), 7α-hydroxydehydroabietic acid (**76235-98-8**), 7β-hydroxydehydroabietic acid (**73609-55-9**), 15- hydroxydehydroabietic acid (**54113-95-0**), maingayic acid (**34327-14-5**), 11-hydroxy-13-(1-hydroxy-1-methylethyl)-podocarpa-8,11,13-trien-7-one (**16755-58-1**), 11-hydroxy-12-methoxyabietatriene (**16755-54-7**), methyl trans-communate (**15798-13-7**), and methyl isopimarate (**1686-62-0**).

**Table 5:** Tabulated (M+H)**^+^** ions, empirical formulas, for the bioactive 649C fractions.

Peak number	Retention time (min)	*m/z*	Relative abundance (%)	Empirical formula Δ (ppm)
**Fraction 649C-F9**				
	33	371.0991	1.4	C_16_H_19_O_10_ (4.7)
	39.8	376.2581	15.49	C_23_H_36_O_4_ (−6.8)
	42.1	368.2422	7.55	C_17_H_36_O_8_ (5.2)
	43.4	368.2426	8.19	C_17_H_36_O_8_ (5.9)
	46.5	718.449	5.73	C_37_H_66_O_13_ (−1.1)
	48.2	371.0994	0.92	C_19_H_17_O_7_N (−1.6)
**1**	50.3	**317.2099**, 650.4375	2.89	C_20_H_29_O_3_ (−3.8)
	51.3	637.4457	56.4	C_40_H_61_O_6_ (−1.0)
	60.6	630.504	1.24	C_36_H_70_O_8_ (−4.0)
**Fraction 649C-F13**				
	24.2	608.3806	2.09	C_30_H_56_O_12_ (6.5)
	30.6	371.0994	1.66	C_16_H_19_O_10_ (5.7)
	30.9	371.0993	1.04	C_16_H_19_O_10_ (5.5)
	38.0	384.2368	7.89	C_17_H_36_O_9_ (3.7)
	40.2	384.2374, 782.4683	2.23	C_38_H_70_O_16_ (2.8)
**2**	41.1	376.2583	6.92	C_23_H_36_O_4_ (−6.8)
	41.6	715.4025	6.76	C_40_H_59_O_11_ (−3.8)
	43.5	718.4499	1.39	C_37_H_66_O_13_ (0.2)
	44.9	718.4499	1.01	C_37_H_66_O_13_ (−1.5)
**3**	45.8	301.2152	1.48	C_20_H_29_O_2_ (−3.5)
**4**	47.5	**303.2309**, 605.4541, 750.4388	1.52	C_20_H_31_O_2_ (−3.3)
**5**	47.7	**305.2464**, 609.4851	1.64	C_20_H_33_O_2_ (−3.5)
	49.6	669.4379	36.27	C_40_H_61_O_8_ (2.7)
**6**	50.9	**319.2257**, 659.4258	2.03	C_20_H_31_O_3_ (−3.3)
	51.7	654.4701	1.51	C_37_H_66_O_9_ (0)
	63.5	621.302	6.87	C_43_H_41_O_4_ (3.2)
	64.5	621.3017	4.05	C_43_H_41_O_4_ (2.8)
	67.2	621.3037	2.19	C_36_H_45_O_9_ (−3.4)
	76.3	371.0991	5.02	C_16_H_19_O_10_ (5.0)

ND, formula not determined.

*Percent relative abundance is determined by peak area.

^†^The putative formula is reported for bold ion.

Fraction 649-F13 was more diverse, with 19 ions having greater than 1% relative abundance by LC-MS analysis. As before, a single ion dominates, *m/z* 669.4379 eluting at 49.6 min. There are no putative matches for this ion in Scifinder. However, putative matches were obtained for five other ions from 649C-F13, identified as peaks **2–6** in **Figure 7**. Peak **2** has *m/z* 376.2583 and empirical formula C_23_H_36_O_4_ and corresponds to [5′aS-(5′aα,7′aα,9′α,10′α,12′aα,12′bβ)]-decahydro-2,2,5′,5′,12′b-pentamethyl-spiro[1,3-dioxolane-4,9′(8′H)-[7a,10]methano[7aH]cyclohepta[g][2]benzoxepin]-3′(2′H)-one (CAS #**49817-39-2**). Peak **3** has *m/z* 301.2152 and empirical formula C_20_H_29_O_2_ which corresponds to (5α,9α,10β)-3-oxo-kaur-15-en-17-al (CAS #**878000-26-1**), 18-oxoferruginol (**108904-92-3**), 4-epidehydroabietic acid (**5155-70-4**), and dehydroabietic acid (**1740-19-8**). Peak **4** has *m/z* 303.2309 and empirical formula C_20_H_31_O_2_ and corresponds to callicarpic acid A (CAS #**1130124-82-1**), 1-[2-(3-furanyl)ethyl]-1,3,4,7,8,8a-hexahydro-1,2,5-trimethyl-4a(2*H*)-naphthalenemethanol (**35319-12-1**), isopimaric acid (**5835-26-7**), and cryptopimaric acid (**471-74-9**). Peak **5** has *m/z* 305.2464 and empirical formula C_20_H_33_O_2_ which corresponds to [4a*S*-(4aα,4bβ,8aα,10aβ)]-4a,4b,5,6,7,8,8a,9,10,10a-decahydro-2-(1-hydroxy-1-methylethyl)-4b,8,8-trimethyl-4(1*H*)-phenanthrenone (CAS #**121926-95-2**), [1R-(1α,4aβ,4bα,7α,8α,10aα)]-7-ethenyl-1,2,3,4,4a,4b,5,6,7,8,10,10a-dodecahydro-8-hydroxy-1,4a,7-trimethyl-1-phenanthrenemethanol (**101467-73-6**), 15,16-dihydroisopimaric acid (**5673-36-9**), 15,16-dihydrosandaracopimaric acid (**4807-69-6**), and arachidonic acid (**506-32-1**). Peak **6** has *m/z* 319.2257 and empirical formula C_20_H_31_O_3_ and corresponds to 4-[2-[(1S,4aR,6S,8aR)-1,4,4a,5,6,7,8,8a-octahydro-6-hydroxy-2,5,5,8a-tetramethyl-1-naphthalenyl]ethyl]-2(5H)-furanone (CAS #**2305691-63-6**), 4-[2-[(1S,4aR,6S,8aR)-decahydro-6-hydroxy-5,5,8a-trimethyl-2-methylene-1-naphthalenyl]ethyl]-2(5H)-furanone (**2305691-62-5**), nudiflopene B (**2226250-84-4**), (1S, 4aS, 4bS, 7S, 8S, 10aS)-7-ethenyl-1, 2, 3, 4, 4a, 4b, 5, 6, 7, 8, 10, 10a-dodecahydro-8-hydroxy-1, 4a,7-trimethyl-1-phenanthrenecarboxylic acid (**2137490-87-8**), callilongisin D (**1367097-06-0**), callilongisin C (**1367097-03-7**), callilongisin B (**1367097-00-4**), (1S,2S,8aS)-1,2,3,7,8,8a-hexahydro-8a-hydroxy-1-methyl-2-(1-methylethenyl)-6-(1-methylethyl)-1-naphthalenepropanoic acid (**1123207-97-5**), 12(S)-hydroxycleroda-3, 13-dien-16, 15-olide (**935293-68-8**), 12(S)-hydroxycleroda-3, 13-dien-15,16-olide (**935293-66-6**), 16ξ-hydroxycleroda-3,13-dien-15,16-olide (**141979-19-3**), 7α-hydroxysandaracopimaric acid (**122537-68-2**), [4aS-(4aα,7aα,8aα,9bβ)]-2, 3, 4, 4a, 5, 6, 7, 7a, 8a, 9b-decahydro-7a-(1-hydroxy-1-methylethyl)-4,4,9b-trimethyl-phenanthro[2,3-b]oxiren-9(1H)-one (**121926-98-5**), calliphyllin (**101467-72-5**), and 3-oxoanticopalic acid (**83997-21-1**).

## Discussion

The most active fractions of the *C. americana* leaf extracts exhibited MICs against *C. acnes* isolates at concentrations as low as 0.016 mg ml^−1^, which is 30 times less than the lowest MIC value found for other botanical extracts previously investigated, including Jeju (*Thymus quinquecostatus* Celak., Lamiaceae—MIC of 0.5 mg ml^−1^), Damask Rose (*Rosa* x *damascene* Herrm., Rosaceae—MIC of 2 mg ml^−1^), Duzhong (*Eucommia ulmoides* Oliv., Eucommiaceae—MIC of 0.5 mg ml^−1^), and Maté (*Ilex paraguariensis* A.St.-Hil.—MIC of 1 mg ml^−1^) (Oh et al., 2009; Tsai et al., 2010). In comparison to the growth inhibition activity, the *C. americana* extracts were less effective at the eradication of existing biofilm. Following the result reported by Coenye *et al.*, we included resveratrol as a biofilm eradication control (Coenye et al., 2012). At the highest concentration tested (394 μg ml^−1^), only 21% inhibition was achieved by resveratrol. However, the previous 80% biofilm eradication reported for resveratrol was at a concentration of 3,200 μg ml^−1^.

The cytotoxicity (IC_50_) of the most active fractions, 649C-F9 and 649C-F13, were at concentrations 32 and >128 times greater than that required for *C. acnes* growth inhibition (IC_50_). These results demonstrate the strong potential of *C. americana* fractions to reduce *C. acnes* fitness at a good therapeutic index ratio of mammalian toxicity to antimicrobial activity.

In other studies, bioassay-guided fractionation of the dried leaf, twigs, and fruits of *C. americana* extracts led to the isolation of six clerodane diterpenes (Jones et al., 2007). Flavonoids including genkwanin, 5-hydroxy-7,4′-dimethoxyflavone, and luteolin were also found in these extracts. The leaf essential oil contained lipids [(E)-2-hexenal and 1-octen-3-ol] as well as monoterpenoids (nopinone, α-pinene, and β-pinene), sesquiterpenoids (α-cadinol, caryophyllene oxide, 7-*epi*-α-eudesmol, α-humulene, humulene epoxide II, intermediol, khusinol, valencene, α-selinene, and 7-*epi*-α-selinene), and triperpenoids (euscaphic acid) (Tellez et al., 2000; Kobaisy et al., 2002; Cantrell et al., 2005; Jones et al., 2007). Some compounds were active against human cancer cell lines with a cytotoxic activity below 5 µg ml^−1^. These include 12(*S*),16-dihydroxycleroda-3,13-dien-15,16-olide, 2-formyl-16-hydroxy-3-α-norcleroda-2,13-dien-15,16-olide, genkwanin, 12(*S*)-hydroxycleroda-3,13-dien-16,15-olide, 16-hydroxycleroda-3,13-dien-15,16-olide, and 16-hydroxy-cleroda-3,11(*E*),13-trien-15,16-olide. Intermedeol and callicarpenal were demonstrated to be effective deterrents of *A. stephensi* and *A. aegypti* mosquitos, and clerodane diterpenes are also known to have insect antifeedant properties (Gebbinck et al., 2002). To the best of the authors’ knowledge, this is the first report on the antibacterial activity of *C. americana* extracts against *C. acnes*.

By colonizing the pilosebaceous follicle and inducing the production of reactive oxygen species highly toxic to the cells (Shalita et al., 2011), *C. acnes* exacerbates the inflammation associated with acne vulgaris. *C. acnes* also worsens the symptoms by inducing immune cells to produce pro-inflammatory cytokines including interleukins and tumor necrosis factors (Vowels et al., 1995). Prior studies on ethanolic extracts of rosemary demonstrated a significant reduction of cytokine production *in vitro* and attenuation of swelling and inflammation in a mouse model (Tsai et al., 2010). The minimum inhibitory concentration was 4 mg ml^−1^, which is 250 times more than the lowest MIC reported in the present paper. By effectively reducing *C. acnes* growth and proliferation, *C. americana* leaf extracts may also impact inflammatory processes. Further research is needed to evaluate this aspect of *C. americana* treatments with *in vivo* infection models.

While treating acne with antibiotics, such as erythromycin and clindamycin, was shown to significantly reduce inflammation, extensive use of oral and/or topical antibiotics since the 1960s has led to the emergence of resistant strains (Eady et al., 1989). This underlines the need for alternative compounds for acne therapy. Other factors such as androgens, poor digestion, or smoking habits (Leyden, 1995) are also important in the development and persistence of the disease. Treatments should focus not only on the consequences but also on the causes of acne and could combine cross-acting compounds to reduce sebum production, correct stomach acidity for better digestion, while also reducing inflammation (Yarnell and Abascal, 2006). Previous studies reported the interesting potential of botanical combinations in treatments to reduce acne (Lalla et al., 2001; Kılıç et al., 2019). Our study highlights the potential of *C. americana* leaf extracts to efficiently reduce bacterial proliferation and lessen acne vulgaris symptoms.

## Conclusion

Our work has demonstrated the promising antibacterial potential of *C. americana* constituents against *C. acnes* growth. Fractions 649C-F9 and 649C-F13 exhibited growth inhibitory activity against a panel of 10 C. *acnes* isolates and with a good therapeutic index of 32 and >128, respectively). Future studies should pursue isolation and structural determination of active components, further examination of the efficacy and safety of the compounds, and examine the mechanism of action for these observed antibacterial effects.

## Data Availability Statement

The raw data supporting the conclusions of this manuscript will be made available by the authors, without undue reservation, to any qualified researcher.

## Author Contributions

RP prepared the extracts and fractions and performed HaCaT cytotoxicity experiments. RP and SH performed antibacterial assays. JL performed the chemical analysis of the extracts. CQ designed and directed the study. RP and CQ analyzed the data and wrote the manuscript. All authors read, revised, and approved the final manuscript.

## Funding

This work was supported by faculty development funds from the Emory University School of Medicine, Department of Dermatology and Emory College of Arts and Sciences, Center for the Study of Human Health.

## Conflict of Interest

The authors declare that the research was conducted in the absence of any commercial or financial relationships that could be construed as a potential conflict of interest.
